# Harmonised LUCAS *in-situ* land cover and use database for field surveys from 2006 to 2018 in the European Union

**DOI:** 10.1038/s41597-020-00675-z

**Published:** 2020-10-16

**Authors:** Raphaël d’Andrimont, Momchil Yordanov, Laura Martinez-Sanchez, Beatrice Eiselt, Alessandra Palmieri, Paolo Dominici, Javier Gallego, Hannes Isaak Reuter, Christian Joebges, Guido Lemoine, Marijn van der Velde

**Affiliations:** 1 European Commission Joint Research Centre (JRC), Ispra, Italy; 2 European Commission, Eurostat (ESTAT), Luxembourg, Luxembourg; 3 GOPA Luxembourg, Luxembourg, Luxembourg

**Keywords:** Environmental sciences, Agriculture

## Abstract

Accurately characterizing land surface changes with Earth Observation requires geo-located ground truth. In the European Union (EU), a tri-annual surveyed sample of land cover and land use has been collected since 2006 under the Land Use/Cover Area frame Survey (LUCAS). A total of 1351293 observations at 651780 unique locations for 106 variables along with 5.4 million photos were collected during five LUCAS surveys. Until now, these data have never been harmonised into one database, limiting full exploitation of the information. This paper describes the LUCAS point sampling/surveying methodology, including collection of standard variables such as land cover, environmental parameters, and full resolution landscape and point photos, and then describes the harmonisation process. The resulting harmonised database is the most comprehensive *in-situ* dataset on land cover and use in the EU. The database is valuable for geo-spatial and statistical analysis of land use and land cover change. Furthermore, its potential to provide multi-temporal *in-situ* data will be enhanced by recent computational advances such as deep learning.

## Abstract (summary)

**Table:** 

Measurement(s)	land cover • land use process • land use
Technology Type(s)	field survey
Factor Type(s)	GPS location • year of data collection
Sample Characteristic - Environment	land
Sample Characteristic - Location	Europe

Machine-accessible metadata file describing the reported data: 10.6084/m9.figshare.12841202

## Background & Summary

Accurate, timely, and representative *in-situ* observations across large areas have always been needed to report statistics on land use, land cover, and the environment. Precise geo-located *in-situ* information is also indispensable to train and validate algorithms that characterize the Earth’s surface based on remotely sensed observations. Comprehensive and thematically rich *in-situ* data can lead to better classifiers and more accurate multi-temporal land surface mapping. This is especially true since increasingly frequent and detailed Earth Observations are being made, for instance by the fleet of Sentinel satellites of the EU’s Copernicus program. These developments are opening avenues to better combine classical statistical surveying and Earth Observation (EO) derived products in the domains of land use and land cover change and environmental monitoring (e.g^1^.).

### Historical background

The Land Use/Cover Area frame Survey (LUCAS) in the European Union (EU) was set-up exactly to provide such statistical information^2^. It represents a triennial *in-situ* land cover and land use data collection exercise that extends over the whole of the EU’s territory (https://ec.europa.eu/eurostat/web/lucas). The LUCAS project was implemented following Decision 1445/2000/EC of the European Parliament and of the Council of 22 May 2000 “On the application of area-frame survey and remote-sensing techniques to the agricultural statistics for 1999 to 2003” and has continued since. While the LUCAS survey concept was initiated and tested in 2001 and 2003^3^, it has been restructured in 2006^4^ and then slightly modified to result in the actual survey design^5^. In 2006, Eurostat, the statistical office of the EU, launched a pilot survey project in 11 countries to test the stratified sampling design. The primary focus was on agricultural areas with emphasis given to easily accessible points. Since then Eurostat has carried out LUCAS surveys every three years with the survey design ever evolving, however the LUCAS *core* component (i.e. the identification of the point, and the surveying of specific variables on different aspects of land cover, land use, and land and water management^6^), has remained comparable for all five surveys.

### Survey design summary

LUCAS collects information on land cover and land use, agro-environmental variables, soil, and grassland. The surveys also provide spatial information to analyse the mutual influences between agriculture, environment, and countryside, such as irrigation and land management. The *in-situ* point data is collected according to a harmonised classification with separate land cover and land use codes. Data quality is assured by a regular two-level quality control (i.e. internal and external), in which all points are evaluated by quality controllers (see^7^ for more details). At each LUCAS point, standard variables are collected including land cover, land use, environmental parameters (the so called *micro data*), as well as one downward facing photo of the point (P) and four landscape *photos* in the cardinal compass directions (N, E, S, W). Additionally to the *core* variables collected, other specific *modules* were carried out on demand such as (i) the transect of 250 m to assess transitions of land cover and existing linear features (2009, 2012, 2015), (ii) the topsoil module (2009, 2012 (partly), 2015 and 2018), (iii) the grassland module (2018), and (iv) the Copernicus module collecting the homogeneous and continuous extent of land cover on a 50-m radius (2018)^8^. Due to the specificity of these modules, their corresponding collected data are not included in the data harmonisation presented in this paper. The topsoil module datasets for 2009, 2012 and 2015 were harmonised and documented separately as an open-access dataset of topsoil properties in the EU^9^.

**Fig. 1:**
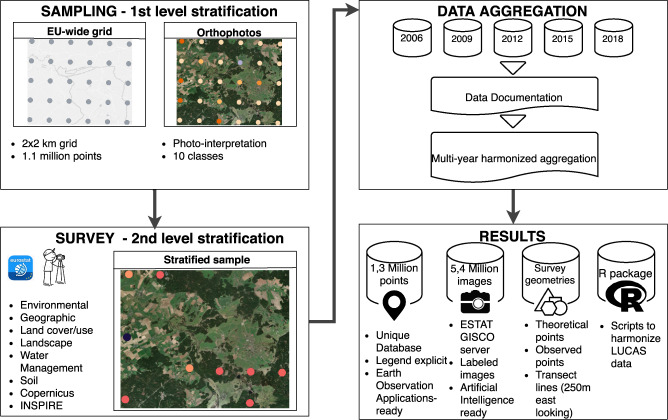
Schematic overview of the LUCAS and harmonisation methodologies. The left side illustrates the sampling at the basis of the production of the LUCAS primary data. The top right side shows the raw base data (micro data). The process of harmonising is contained within the multi-year harmonised aggregation block and is the subject of the following two sections. The bottom right presents the four main outputs associated with this manuscript (more in section Data Records) - a harmonised, legend-explicit, multi-year, ready-to-use, version of the LUCAS micro data (section *Overview of multi-year harmonised LUCAS survey database*^49^,), a database with all cardinal-direction landscape and point photos collected during the surveys, including their respective EXIF attributes (section Overview of EXIF photos database, EXIF table^49^, photos on https://gisco-services.ec.europa.eu/lucas/photos/, the survey geometries^49^ and a R package to generate the data^51^.

LUCAS is a two phase sample survey. The LUCAS first phase sample is a systematic selection of points on a grid with a 2 km spacing in Eastings and Northings covering the whole of the EU’s territory^10^. Currently, it includes around 1.1 million points (Fig. 1) and is referred to as the master sample. Each point of the first phase sample is classified in one of ten land-cover classes via visual interpretation of ortho-photos or satellite images^11^. The core sampling and survey methods have remained the same throughout the five surveys. Nevertheless evolving goals of the surveys have led to slightly different sample point allocations for different land covers. In 2006, the main objective was to “make early estimates of the main crop areas”, along with the ability to collect information on agri-environmental indicators in the context of the monitoring of the Common Agriculture Policy (CAP)^3^. In 2009, the main objective was to estimate areas, especially in conjunction with other data sources such as Corine Land Cover (CLC)^10^. In 2018, the main objective was to “monitor social and economic use of land as well as ecosystems and biodiversity”^5^. Additionally, in 2018, a linear logistic regression model based on LUCAS 2015 and 16 additional variables were used as co-variates to forecast the most probable land cover for each of these points^5^. From this stratified first phase sample, the second phase sample of points is selected to obtain the desired statistically representative spatial distribution of sampled land cover classes according to the first phase visual interpretation. With LUCAS 2018 this amounts to 337845 points, out of which approximately 240000 points are visited in the field by surveyors to collect additional information that cannot be assessed remotely.

The *in-situ* nature of the survey implies that the majority of the data are gathered through direct observations made by surveyors on the ground. Those points which are unlikely to change and points which are too difficult to access are classified by photo-interpretation in the office, using the latest available ortho-photos or Very High Resolution (VHR) images. Although most of the points a-priori assigned for *in-situ* assessment can effectively be visited in the field, those that cannot be reached, because of lack of access to the point or the point location being at more than 30 minutes walking distance from the closest point reachable by car. Those points are thus photo-interpreted on ortho-photos or Very High Resolution (VHR) images in the field by the field surveyor. Furthermore, sometimes a significant difference exists between the theoretical LUCAS point and the actual GPS location reached by the surveyor. Observations are collected for the LUCAS point, while the photos are taken at the actual GPS location. Both locations and the distance between them is noted down.

### Previous LUCAS use cases and shortcomings

In the scientific literature, LUCAS land cover and land use survey data have been used to derive statistical estimates^2^, to describe land cover/use diversity at regional level^12^, and its sampling frame was used as a basis for various applications including assessing the availability of crowd-sourced photos potentially relevant for crop monitoring across the EU^13^. LUCAS was designed to derive statistics for area estimation (e.g^3^. and^10^). Recently, several researchers have started to use LUCAS data in large scale land cover mapping processes, especially as a source of training and/or validation data for supervised classification approaches at regional/national scale^14–20^.

Several drawbacks become apparent when working with the original LUCAS datasets. While the inconsistencies could be due to the enumerators’ subjectivity in interpretation of the legends and the legend itself, it is also related to the complexity of the field survey: large number of surveyors (>700), complex documentation for the enumerators (>400 pages combining all the documents), translated to 20 languages. These drawbacks hinder the further use of the LUCAS data by the scientific community as a whole and in particular by users who are active in emerging fields of big data analytics, data fusion, and computer vision. Such drawbacks include:

- Inconsistencies and errors between legends and labels from one LUCAS survey to the next which is hampering temporal analysis.
- Missing internal cross-references in the datasets that would facilitate computation and linking observed variables, photos, etc.
- The original full resolution photos taken at each surveyed point are not available for download.
- The lack of a single-entry point or consolidated database hampering automated processing and big data analysis.

Therefore, we have gone through an extensive process of cleaning by semantic and topological harmonisation, along with connecting the originally disjoint LUCAS datasets in one consolidated database with hard-coded links to the full-resolution photos, openly accessible along with this paper.

## Methods

Having contextualized the LUCAS survey, we proceed with describing the full methodological workflow to harmonise the data, as schematically shown in Fig. 1. The Sampling and Survey sub-figures provide an overview of the methodological framework of the LUCAS data collection itself (see previous section *Background & Summary*). The Data aggregation and Results sub-figures illustrate the work carried out in this study. The datasets collected during the five surveys (in 2006, 2009, 2012, 2015, 2018) are the main LUCAS products available (more in section *Micro data collection and documentation (Protocol 1)*). These datasets and their respective data documentation were used to create the multi-year harmonised database. The harmonisation process is described below and in Table 1. Associated with the summary Table 1, the Table 2 provides name changes, the Table 3 provides the new columns added, the Online-only Table 1 provides the missing column adding and the Online-only Table 2 provides the variable re-coding. The results are consolidated in one single consistent and legend-explicit table along with hard-coded links to the full resolution photos (stored on the GISCO, https://gisco-services.ec.europa.eu/lucas/photos/). The LUCAS primary data includes alpha-numerical variables and field photographs linked to the geo-referenced points.

**Table 1:** Aggregation of micro data - summarizing the different steps applied to harmonise the survey data.

Source	Year	Points (n)	Protocol 1	Protocol 2
^21^	2006	168401	Data download, Data documentation^37,40^, Preparation (year aggregation), Generate mapping files	Column renaming (Table 2), Missing column adding (Online-only Table 1), New column adding (Table 3), Character case uniformity, Variable re-coding (Online-only Table 2), Column order
^33^	2009	234623	Data download, Data documentation^38,41^, Preparation, Generate mapping files	Column renaming (Table 2), Missing column adding (Online-only Table 1), New column adding (Table 3), Character case uniformity, Variable re-coding (Online-only Table 2), Column order
^34^	2012	270272	Data download, Data documentation^39,42^, Preparation, Generate mapping files	Column renaming (Table 2), Missing column adding (Online-only Table 1), New column adding (Table 3), Character case uniformity, Variable re-coding (Online-only Table 2), Column order
^35^	2015	340143	Data download, Data documentation^46^, Preparation, Generate mapping files	Column renaming (Table 2), Missing column adding (Online-only Table 1), New column adding (Table 3), Character case uniformity, Variable re-coding (Online-only Table 2), Column order
^36^	2018	337854	Data download, Data documentation^47^, Preparation, Generate mapping files	Missing column adding (Online-only Table 1), New column adding (Table 3), Character case uniformity

**Table 2:** Table of renamed variables.

Year added to	Old name	New name
2006	surv_date	surveydate
2006	x_laea	th_lat
2006	y_laea	th_long
2009, 2012, 2015	area_size	parcel_area_ha
2009, 2012, 2015	date	surveydate
2009, 2012, 2015	lc1_pct	lc1_perc
2009, 2012, 2015	lc2_pct	lc2_perc
2009, 2012, 2015	lc1_species	lc1_spec
2009, 2012, 2015	lc2_species	lc2_spec
2009, 2012, 2015	land_mngt	grazing
2009, 2012, 2015	obs_dir	obs_direct
2009, 2012, 2015	photo_e	photo_east
2009, 2012, 2015	photo_w	photo_west
2009, 20122009, 2012, 2015	photo_n	photo_north
2009, 2012, 2015	photo_s	photo_south
2009, 2012, 2015	photo_p	photo_point
2009, 2012, 2015	tree_height_surv	tree_height_survey
2009, 2012, 2015	soil_stones	soil_stones_perc
2012, 2015	tree_height_mat	tree_height_maturity
2015	lu1_pct	lu1_perc
2015	lu2_pct	lu2_perc
2015	protected_area	special_status
2015	pi_extension	office_pi

**Table 3:** Table of newly added columns.

Column name	Description
letter_group	First level of LUCAS LC1/2 classification
year	Year of the survey
file_path_gisco_n/s/e/w/p	Path to cardinal or point photo on GISCO
th_geom	Geometry of theoretical LUCAS point according to grid
gps_geom	Geomtery at the point the surveyor reached
th_gps_dist	Calculated distance between the two points
visit	Numbers of years of visit for the LUCAS point

### Micro data collection and documentation (Protocol 1)

The first step is to collect the data from the source for each survey year (see Table 1 Source). The raw micro data for the harmonised database presented here are the five LUCAS campaigns, which can be downloaded from the official Eurostat website (https://ec.europa.eu/eurostat/web/lucas). The LUCAS micro data for 2006^21^ is divided into a separate file for each of the 11 surveyed countries (Belgium^22^, Czechia^23^, Germany^24^, Spain^25^, France^26^, Italy^27^, Luxembourg^28^, Hungary^29^, Netherlands^30^, Poland^31^, and Slovakia^32^). The LUCAS micro data is provided aggregated for all countries for every other survey years, whereby the data can also be downloaded separately by country (2009^33^, 2012^34^, 2015^35^) in CSV format and 2018^36^ in 7z zipped format. The second step is to collect the documentation that facilitates translating the alpha-numerical class-description in the original datasets into explicit information. For 2006, 2009 and 2012, the survey data comes with a content descriptor (2006^37^, 2009^38^, 2012^39^), though the content descriptor doesn’t necessarily have the same number of variables as the data; and the variables themselves sometimes have a slightly different name. These inconsistencies were resolved with assistance from the technical documents (LC1 (Instructions, 2006^40^, 2009^41^, 2012^42^) and LC3 (Classification, 2006^43^, 2009^44^, 2012^45^). From 2015 and 2018, the data is provided with a record descriptor (2015^46^, 2018^47^), which contains information on variable name, data type and description in a more consolidated fashion, making it easier to find information about the relevant variable.

The third and final step in Protocol 1 is the generation of the mapping files used for value recoding. The workflow maps the ascertained relationship between those variables that are the same but have changed in name or alpha-coding between surveys. To recode all variables coherently from one survey to the next, the original data is changed permanently. All transformations are done by recoding ordinal variables to be compliant with the encoding of variables used in the last survey (2018). These mappings serve as a blueprint for the transformation and data integration described in Protocol 2.

### Micro data harmonisation (Protocol 2)

**Fig. 2:**
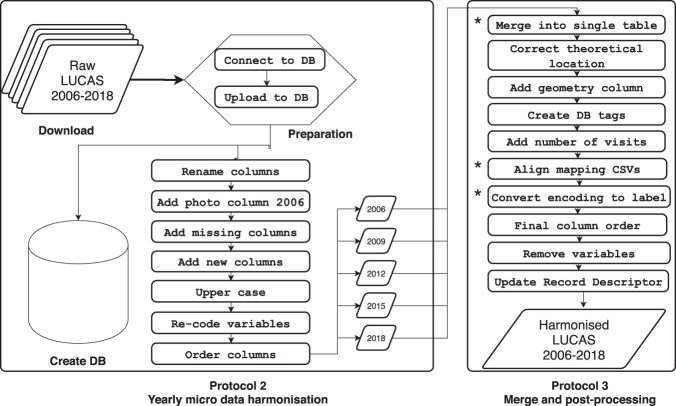
Processing workflow to harmonise the survey data. Asterix used to indicate steps after which there is a performed consistency check (*Merge into single table*, *Align mapping CSVs*, and *Convert encoding to label*).

The harmonisation workflow, alongside the performed database consistency checks, is shown in Fig. 2 and the code is described in code section (section *Code availability*). The general principle of the harmonisation workflow was to convert all the field legends to fit with the latest i.e. the 2018 database layout (the next LUCAS is planned for 2022).

Some notable changes in the source tables had to be made in order to make the harmonisation and subsequent merger into one complete table possible. This was accomplished with the above-mentioned instance-mapping files (Section Micro data collection and documentation (Protocol 1)). All manipulations executed over the separate tables prior to the merger are listed in Table 1 under heading ‘Protocol 2’:

1. Rename columns - iteratively renaming columns to align them with the last (in this case 2018) survey. Performed on all tables but 2018 by using the Rename_cols() function from the package.
2. Add photo column 2006 - adds columns *photo_north*, *photo_south*, *photo_east*, *photo_west*, and *photo_point* on account of them missing from the 2006 base data. Adding is done by cross-referencing the EXIF picture database (see section Overview of EXIF photos database). Performed solely on table for 2006 by using the Add_photo_field_2006() function.
3. Add Missing columns - iteratively adding all columns that are present in one table and not present in the others. Performed on all tables by using the Add_missing_cols() function.
4. Add new columns - iteratively adding all newly created columns. These include the variables ‘letter group’, ‘year’, and ‘file_path_gisco_n/s/e/w/p’ (for more information check Online-only Table 3). Performed on all tables using the Add_new_cols() function.
5. Upper case - iteratively converting all characters of selected fields to upper case. Performed on all tables using the Upper_case() function.
6. Re-code variable - iteratively re-coding selected variables according to created mapping CSV files, designed referring back to the reference documents. Performed on all tables but 2018 by using the Recode_vars() function.
7. Order columns - iteratively ordering all columns according to the template from the 2018 survey. Performed on all tables but 2018 by using the Order_cols() function.

### Merge and post-processing (Protocol 3)

The third part of the harmonisation process includes the merging of the harmonised tables of each survey year plus additional steps listed below before exporting the final data outputs.

1. Merge into single table - Merge the five harmonised tables to one unique table via Merge_harmo() function. Consistency check performed after this successful execution on newly generated Table.
2. Correct theoretical location - Applying a correction of the values of columns *th_long* and *th_lat* for merged harmonised table according to the latest LUCAS grid via the Correct_th_loc() function.
3. Add geometry columns - Location of theoretical point(*th_geom*), location of lucas survey (*gps_geom*), lucas transect geometries (*trans_geom*) and distance between theoretical and survey point (*th_gps_dist*). Done by the Add_geom() function.
4. Create database tags - Primary key, index, and spatial index via the Create_tags() function.
5. Add number of visits column - column to show the number of times between the years when the point was visited thanks to the Add_num_visits() function.
6. Align mapping CSVs - Corrects any typo, spelling mistake, or spelling difference in the user-created mapping CSVs, used to generate labels in subsequent function that converts encoding to label by aligning them to the mapping CSV of the latest survey. Done by the Align_map_CSVs() function. Consistency check performed after this successful execution on newly generated mapping CSVs.
7. Convert encoding to label - Create columns with labels for coded variables and decodes all variables where possible to explicit labels. Performed with User_friendly() function. Consistency check performed after this successful execution.
8. Final column order - Re-order columns of final tables with the Final_order_cols() function.
9. Remove variables - optional function to remove variables which the technician deems not necessary for the new harmonised product. Done with the Remove_vars() function.
10. Update record descriptor - Updates Record descriptor by adding a field (*year*) showing the year for which the variable exists and removing variables listed in the optional function for removing variables from record descriptor. Done with the Update_RD() function.

The workflow ends with the output exports. The table is exported as CSV and the geometries as shapefiles. The full workflow is dependent on two software prerequisites. Firstly, one must have a running PostgreSQL server, and secondly, an installation of R (more about the versions used in section Code availability). The pipeline is provided as a R package for ease of reproducibility and transparency (section Code availability).

### Full resolution LUCAS photos

In addition to the alphanumerical and geometry information of the survey, a complete database with full-resolution point and landscape photos was set up with photos retrieved from Eurostat. This archive was organised as a table with all the exchangeable image file (EXIF) variables for each of the images, among which a unique file path, as stored on the Eurostat GISCO server for easy retrieval by other researchers. Besides the EXIF attributes, each photo is also hard-coded with the respective point ID of the LUCAS point and the year of survey. The photos’ metadata were extracted with ExifTool (v 10.8)^48^ resulting in a database of photos that was compared for completeness with the survey data records. The hard-coded HTTPS links to each photo in the consolidated database allow for large data volume queries and selection tasks.

## Data Records

The first section *Storage* describes each data-set provided along with this manuscript including the table, photo, and geometry databases along with the R package created to compile and construct all the data. The second section *Overview of multi-year harmonised LUCAS survey database* provides an outline of the resulting harmonised database and the last section *Overview of EXIF photos database* provides an overview of the photo database.

### Storage

1. **Multi-year harmonised LUCAS survey data**. The harmonised database (available for download here^49^ and also archived as compressed folder here^50^) contains 106 variables and 1351293 records corresponding to a unique combination of survey year and field location. The same dataset is also available for each year with a different file for users interested only in one specific survey. The database is provided with a **Record descriptor** (Online-only Table 3 presents a summary, the complete table is available here in CSV format^49^ in the supporting files). This record descriptor specifies variable name, data type, range of possible values and meanings. In the documentation one can find more information about the variable and a short description, along with comments concerning the variable that the authors have deemed important. Additionally, the tables in **LUCAS-Variable and Classification Changes**, in the supporting files, contain documentation for users to quickly identify the differences between LUCAS campaigns and the harmonised database. The file contains four tables:

1. “References”: Description and a legend of the used colors of the different tables;
2. “Harmonised DB”: a comparison of all the collected variables of the 2018 survey with the variables of the harmonised database and an overview of the actions to harmonise the data;
3. “Variable changes”: an overview/ comparison of all collected variables between all campaigns from 2009 to 2018 highlighting the changes;
4. “LC (LU) changes”: an overview of the possible LC and LU codes of each campaign highlighting the changes.

2. **LUCAS survey geometries/point locations**. To facilitate spatial analysis and usability, three types of geometries are provided as distinct shapefiles (see the geometries folder downloadable on^49^):

1. LUCAS theoretical points (*th_long*, *th_lat*),
2. LUCAS observed points (*gps_lon*, *gps_lat*) and
3. LUCAS transect lines (250-m east looking lines).

3. **High resolution LUCAS photo archive**. The 5.4 millions of photos collected during the five surveys are available at https://gisco-services.ec.europa.eu/lucas/photos/. For each *in-situ* point, landscape (N, E, S, W), and point (P) photos are available. The EXIF information of all the photos were extracted and are provided as an additional table (*lucas_harmo_exif.csv*^49^).
4. **R package**. The scripts to harmonise the LUCAS data is provided as an open source R package along with the documentation^51^.

### Overview of multi-year harmonised LUCAS survey database

Among the data provided with the current study described in the previous section, the multi-year harmonised LUCAS survey database contains the five LUCAS surveys, i.e. a total of 1351293 observations that have been made at 651676 unique locations (Table 4). The total number of surveyed points has increased significantly from the 2006 pilot study (168401) to 2015 (340143) (Table 4). This rise is mainly due to the increase in terms of thematic richness, scope, and scale of the study from what was primarily an evaluation of agricultural areas (2006) to a more holistic and exhaustive inspection of the EU territory. Further, the total number of surveyed countries increased from 11 in 2006 to 28 in 2018 (Table 4). Over the five surveys, 1 031 813 observations (76.36%) were done *in-situ*. Out of these *in-situ* observations, 94% have been surveyed within 100 m distance of the theoretical LUCAS point and 6% were more than 100 m away from the point. The proportion of points where actual *in-situ* data was collected has decreased from 92.18% in 2006 to 63.67% in 2018. Furthermore, 10.92% of the points (i.e. 147574) that were visited *in-situ* turned out not be accessible in practice and are photo-interpreted in the field. The number of points surveyed per country and per year ranged between 79 (Malta) to 48215 (France). Finally, over the five surveys, 1677 points were out of national territory, i.e. “NOT EU” corresponding to water outside national borders or countries including Russia, Turkey, Albania and Switzerland).

**Table 4:** Number of LUCAS points per country and per year.

	2006	2009	2012	2015	2018	Total #
**AT**	—	4961	6469	8839	8840	29109
**BE**	2370	1804	2446	2899	3659	13178
**BG**	—	—	6641	7677	7678	21996
**CY**	—	—	1442	1726	2313	5481
**CZ**	5626	4662	5514	5712	5713	27227
**DE**	27507	21113	24939	26598	26777	126934
**DK**	—	2540	3442	3665	3703	13350
**EE**	—	2663	2200	2637	2665	10165
**EL**	—	7758	7821	12521	12622	40722
**ES**	34489	29912	35377	50281	45314	195373
**FI**	—	19895	13476	16116	16182	65669
**FR**	39070	32318	38324	48188	48215	206115
**HR**	—	—	—	3532	4239	7771
**HU**	8422	5513	4637	5169	5514	29255
**IE**	—	4164	3484	4907	4975	17530
**IT**	20291	17790	20985	28693	28294	116053
**LT**	—	3860	3889	4505	4584	16838
**LU**	197	152	213	251	340	1153
**LV**	—	3825	4420	5374	5376	18995
**MT**	—	—	79	79	79	237
**NL**	2916	2449	2237	2521	5011	15134
**PL**	24128	18487	21797	22980	23086	110478
**PT**	—	5423	7332	9006	7168	28929
**RO**	—	—	14278	16720	16725	47723
**SE**	—	26656	22420	26648	26709	102433
**SI**	—	1203	1621	1923	1922	6669
**SK**	3385	2898	2455	2755	2898	14391
**UK**	—	14438	12214	16803	17253	60708
**NOT EU**	—	139	120	1418	—	1677
**Total # records**	168401	234623	270272	340143	337854	1351293
**Total # countries**	11	23	27	28	28	—
***In-situ*** #	155238	175029	243603	242823	215120	1031813
***In-situ*** **[%]**	92.18	74.6	90.13	71.39	63.67	76.36
***In-situ*** **PI #**	13163	59594	26669	25254	22894	147574
*In-situ* **PI [%]**	7.82	25.4	9.87	7.42	6.78	10.92
**Office PI #**	—	—	—	71970	99803	171773
**Office PI [%]**	—	—	—	21.16	29.54	12.71
**Other #**	—	—	—	96	37	133
**Other [%]**	—	—	—	0.03	0.01	0.01

The total number of records is provided by year and also split according to the type of observation: *In-situ* (direct observation), *In-situ* PI (*In-situ* Photo-Interpreted if point is not accessible) or Office PI (Photo-Interpreted in the office and thus not *in-situ*).

**Fig. 3:**
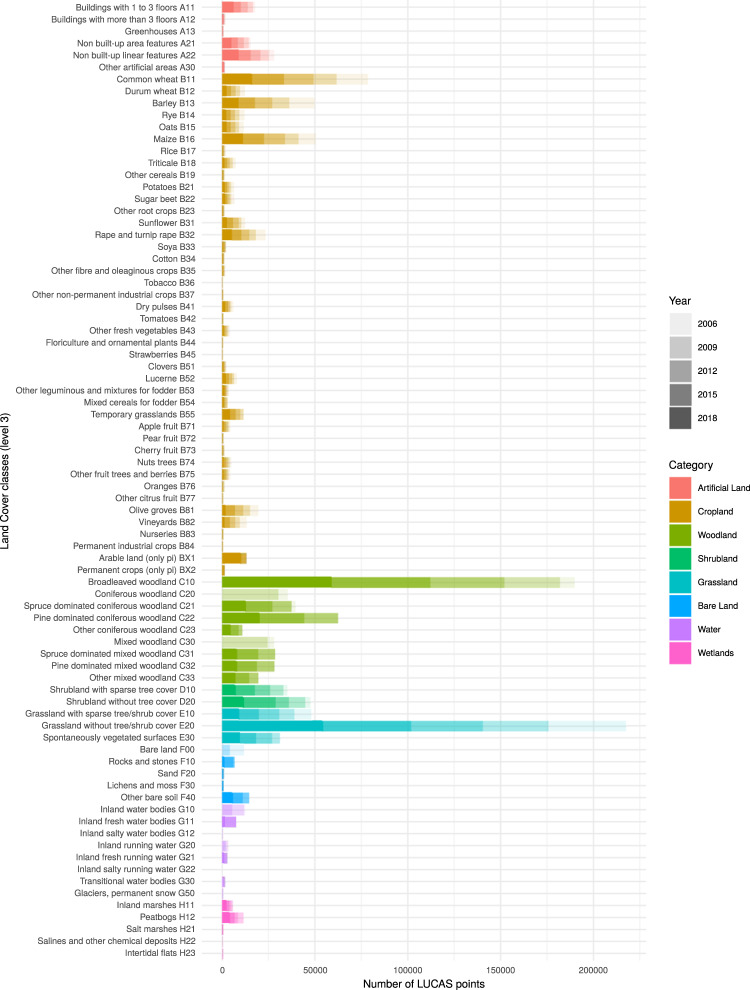
Distribution of land cover classes in the multi-year harmonised LUCAS database. In cases where survey years are not present please orientate oneself with reference to adjacent classes of the same color. Counting for the distribution of each class begins at 2018 and ends with 2006 due to the relative abundance of 2018 in terms of classes compared to other years.

Figure 3 provides the accumulative frequency of assigned level-3 classes (out of 77 classes in total) to the surveyed points, sorted by reference year. Land Cover/Land Use (LC/LU) classification specifications can be found in the new reference document, containing the harmonised C3 legend (see Harmonized C3 legend in^49^).

The classification system follows rules on spatial and temporal consistency - it can be applied and compared both between locations in the EU and by survey years. Additionally, excluding 2006, it is ‘as much as possible’ compatible with other existing LC/LU systems (e.g. Food and Agriculture Organization (FAO), statistical classification of economic activities in the European Community (NACE) (2009–2018) and fulfills the specifications of the European Infrastructure for Spatial Information in Europe (INSPIRE) (2015-2018)). To inform about changes in two consecutive surveys, the data providers describe the adjustments to the terminology in the documentation. The 3-level legend system is arranged hierarchically, whereby the first level (letter group) corresponds to the eight main classes obtained by ortho-photo-interpretation during the second level stratification phase (Fig. 1); the second and third level, representing subcategories of these main classes are indicated by a combination of the letter group and further digits.

**Fig. 4:**
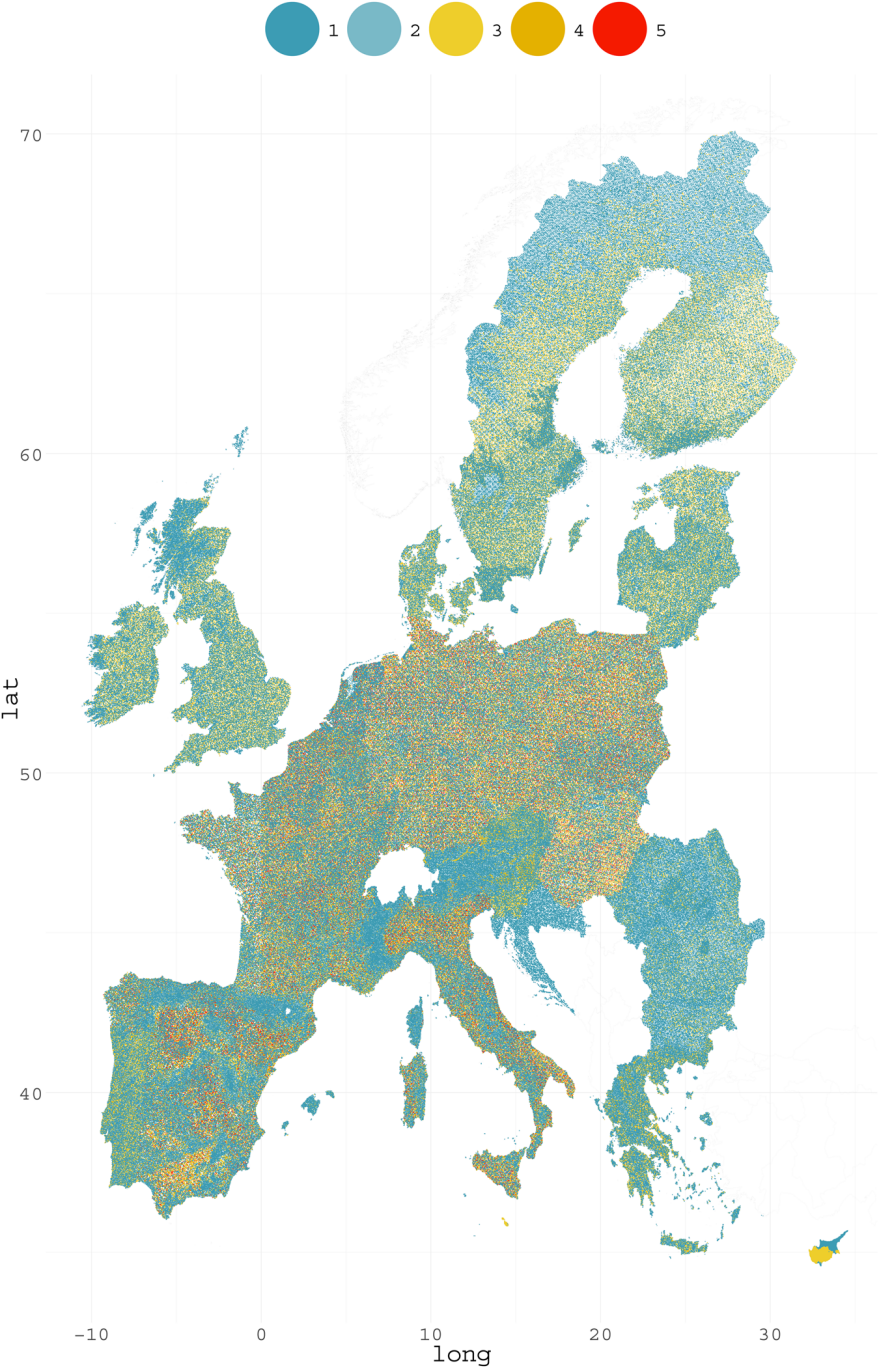
Number of visits to each LUCAS survey point over the five surveys between 2006 and 2018, 651780 points were at least surveyed once. Visit ranges from one to five.

The number of point visits is shown in Table 5. Some LUCAS points were visited once in 15 years (n = 332605) while others were visited each time, thus totaling five visits (n = 35204). This means that 651780 locations were at least visited once. Figure 4 shows a map with the visit frequency for each point over Europe.

**Table 5:** Number of LUCAS points and visits.

Frequency of point visits	1	2	3	4	5
**LUCAS points (n)**	332605	101052	91112	91807	35204

### Overview of EXIF photos database

The available photos (N, E, S, W, P, i.e. North, East, South, West, and Point) were catalogued totaling 5440459 photos for the 5 surveys (see Table 6 for detailed distribution). The *lucas_harmo_exif.csv* table contains the essential and available LUCAS EXIF information (27 variables) for all the photos 2006, 2009, 2012, 2015, 2018. While the observation location is recorded by the surveyor during the LUCAS field survey (*gps_lon*, *gps_lat*), the digital cameras with GPS could also capture the location where the photos were taken as well as the orientation, *i.e*. the azimuth angle. In the first surveys, the digital camera and the GPS were separate devices. The orientation was determined with a traditional compass. The data were used to cross-validate the geo-location reported during the survey. To assess the availability of this information, the EXIF information of the 5440459 photos was retrieved. As summarised in the two last columns of Table 6, the photos with geo-location information have increased considerably through time, i.e. 0% in 2006, 5.4% in 2009, 34.2% in 2012, 68.5% in 2015 and finally 72.9% in 2018. For azimuth angle, there is no information on orientation for the photos taken in 2006 and 2009. However, respectively 15.3%, 22%, and 6.7% of the photos have EXIF orientation information for 2012, 2015, and 2018.

**Table 6:** Number of LUCAS photos per year, per type (N, E, S, W, P) with proportions that have EXIF geo-location (Location [%]) and orientation information (Orientation [%]).

Year	East	North	Point	South	West	TOTAL	Location [%]	Orientation [%]
**2006**	137461	137426	134538	137368	137179	683972	0	0
**2009**	199208	199264	171165	199129	199117	967883	5.4	0
**2012**	269329	269286	243074	269277	269205	1320171	34.2	15.3
**2015**	265421	265392	242772	265368	265285	1304238	68.5	22
**2018**	237259	237529	215190	237262	236955	1164195	72.9	6.7
**Total**	**1108678**	**1108897**	**1006739**	**1108404**	**1107741**	**5440459**		

**Fig. 5:**
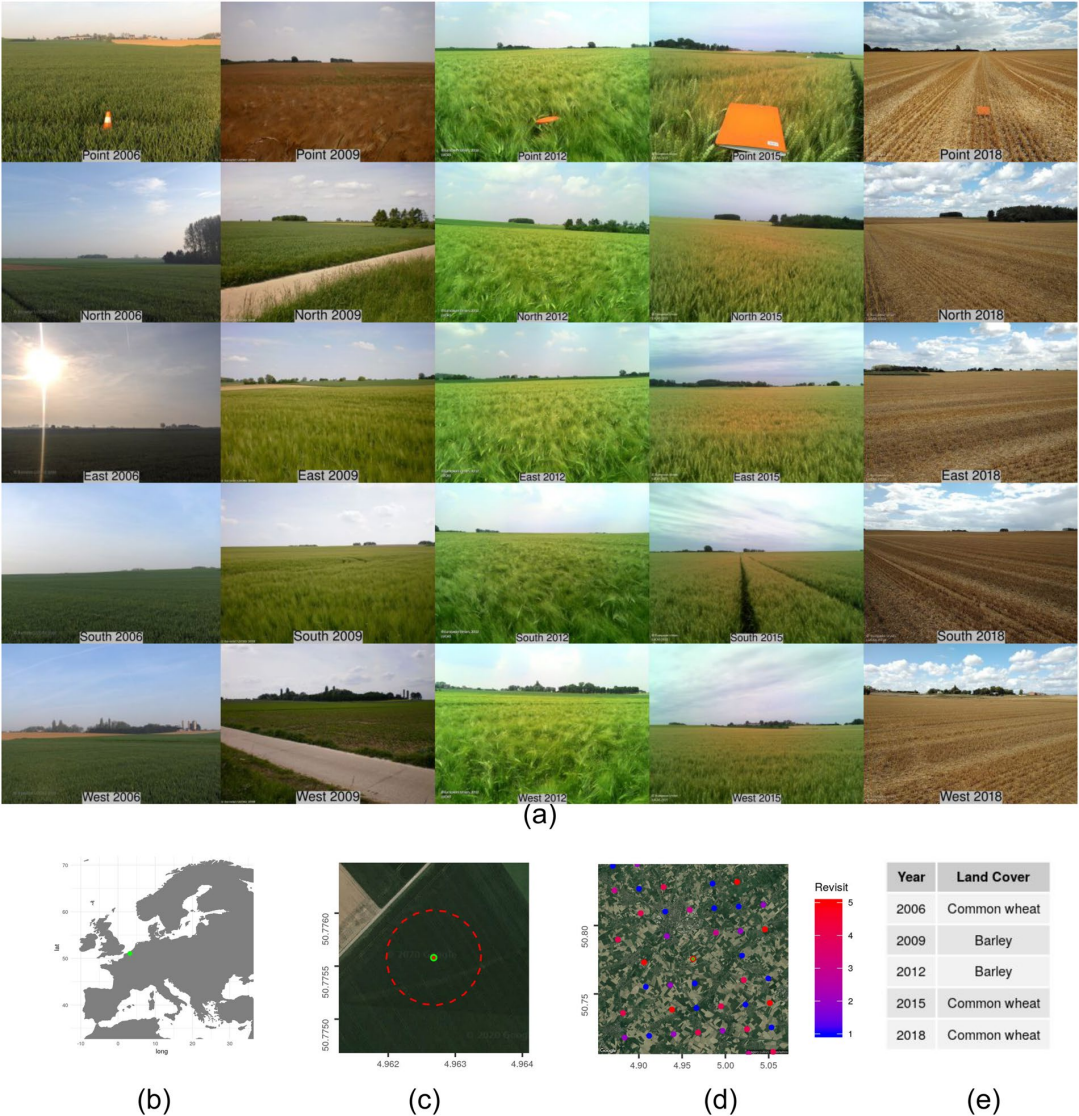
Overview of the data available for a LUCAS point that was visited five times: (**a**) Point, North, East, South and West photos for 2006, 2009, 2012, 2015 and 2018, (**b**) Location of the point in the EU, (**c**) Zoom showing the point (3-m diameter in green, 50-m diameter in dashed red), (**d**) Visit frequency on a 20 by 20 km square centered on the point, and (**e**) *In-situ* land cover observation of the point for the different years.

Each point surveyed has potentially five photos (N, E, S, W, P) per surveyed year (Fig. 5(a)). The EXIF table database is a table of records, corresponding to the photos taken in the cardinal orientations plus the point for each one of the points for the five surveys. The table holds information on the point ID, year of survey, path to the full resolution image and an wide variety of EXIF attributes, including coordinates, orientation, camera model, exact time and date, Eurostat metadata, etc.

It was decided that having this information in a separate table is more sensible in terms of storage size and accessibility, whereby cross-table checks can easily be performed by executing joins between the tables based on point ID and year of survey. By combining this information from the two tables (i.e. the multi-year harmonised LUCAS survey database and the EXIF table database) one arrives at a significantly large set of labeled examples, corresponding to images of the 77 different types of recorded land cover.The background RGB imagery for (c) and (d) is obtained from “Map data ©2019 Google”.

## Technical Validation

The first part of this section briefly summarises the LUCAS field surveys quality check. The section then focuses on analyses carried out specifically to support the technical quality of the multi-year harmonised LUCAS database process.

The LUCAS surveyed observations are subject to detailed quality checks (see LUCAS metadata^52^ and the data quality control documents available for 2009^53^, 2012^54^, 2015^55^). First, an automated quality check verifies the completeness and consistency after field collection. Second, all surveyed points are checked visually at the offices responsible for collection. Third, an independent quality controller interactively checks 33% of the points for accuracy and compliance against pre-defined quality requirements, including the first 20% observations for each surveyor, to prevent systematic errors during the early collection phase.

The presented data consolidation effort seeks to enhance the quality of an existing product. Ensuring data quality by harmonisation throughout the years is thus essential. Data quality was ensured by taking into account validity, accuracy, completeness, consistency, and uniformity throughout data processing (Fig. 2):

- **Validity** of the harmonised database was ensured via data type (for which information can be found in the record descriptor) and a unique constraint of a composite key (consisting of the point ID and year of survey).
- **Accuracy** of the data relies on the source data for which the quality was assessed as described in the previous paragraphs.
- **Completeness** checking shows that since several variables have been added over the years, many missing values exist. In such cases, fields were populated with null values. Consistency across surveys has been enhanced. All surveys were harmonised towards the 2018 survey.
- **Consistency** of the presented dataset was internally ensured through running checks at various stages of processing.
- **Uniformity** checks revealed that the geographical coordinates in columns *th_long* and *th_lat* show different locations between some survey years. In the interest of complete uniformity, it was decided to have the values of these variables hard coded from the LUCAS grid. Because the LUCAS grid is a non-changing feature of all LUCAS surveys, the location of each point remains the same throughout the years. Thus any discrepancy between the recorded theoretical location of a LUCAS point in the micro data and the grid must be corrected. This was done for all but 64 points from 2006 which where recorded on an inaccurate location and were thus removed from the grid.

**Fig. 6:**
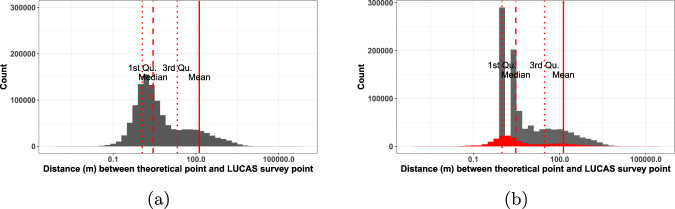
Comparison of distributions between (**a**) calculated distances and (**b**) surveyed distances between LUCAS theoretical points and actual GPS position of surveyor. The red-colored part of the distribution in subfigure (**b**) represents the data from 2006, which is copied from the calculated distances (*th_gps_dist*).

**Fig. 7:**
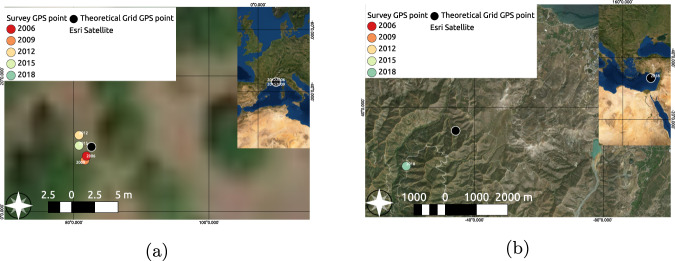
Stability of points and location change over time as illustrated: (**a**) Example of a surveyed point (id 40402278) at close distance (<2 m) and (**b**) Example of a surveyed point (id 63861648) at large distance (1938m). Location change can be either because of survey conditions, the accessibility of the terrain, and/or accuracy of GPS positioning. The background RGB imagery is obtained from “Map data ©2019 Google”.

To further asses spatial accuracy of the data, the distance between the theoretical point from the LUCAS grid (*th_long*, *th_lat*), and the actual GPS measurement of the survey observation point (*gps_lon*, *gps_lat*) were compared. This is important for several reasons - firstly, it allows to ascertain the real distance between the point actually surveyed and the point supposed to be surveyed, which is, in a sense, a proxy for the quality of the surveyed observation itself; secondly, it is an accuracy check of the surveyed distance between the theoretical point and the survey observation point, as collected by the surveyor, “as provided by the GPS (in m)” (column *obs_dist*), and the distance between the same points as calculated from the data (column *th_gps_dist*). It must be noted that for the 2006 survey the variable *obs_dist* was collected as a range, whereas for the other years it represents the actual value of the distance. Because of this lack of uniformity, it was decided to hard code the values for 2006 to match exactly with the calculated distance. In this way we ensure consistency in the data type of the column, yet sacrifice the nuances from changing the original data. The procedure explains that, in 2006, we see a 100% match between recorded and calculated distance (Table 7), whereby for 2009 a match of 96.3%, meaning that for only 3.7% of the cases did the value not match. In carrying out this comparison it became apparent that the percentage of matching distances has increased throughout years probably due to better precision of positioning sensors. Thus the total amount of error in 2018 is reduced to a negligible 0.31%. Furthermore, the comparison was instrumental in the flagging and removing of a number of records that have inaccurate GPS coordinates most probably due to sensor malfunction. Cross-checking with the source data, we found that the error is indeed present in the source data, rather than introduced during processing - something which would have been hard to spot otherwise. The distribution of these calculated distances, alongside an equivalent distribution of the surveyed distances, can be found in Fig. 6. The distance between 75% of the points (1–3 quantile) is between 1.1 and 21.2 meters, meaning that only a fourth of the points have a distance greater than this. For the surveyed distances the ranges are similar - 75% of the values fall between 1.0 and 30.0 meters. From the distributions we see that there is a lot more nuance in the values of the calculated distances, which makes sense as they are represented by numbers with decimals, which have a lower frequency than the integers, representing the surveyed distances. The values shown in the red part of the histogram of surveyed distances represent the values from 2006, which are copied from the calculated distance in order to hard code a numerical in the place of the categorical value of the variable in the source data. The theoretical grid of LUCAS point location is stable over time. However, according to the survey conditions and the terrain and accuracy of the GPS positioning, the surveyor may not be able to reach the point. This results in effective variations of the position of the observer through time (Fig. 7).

**Table 7:** Percentage (%) of points for which the distances between the theoretical point from the LUCAS grid (*th_long*, *th_lat*) and the actual GPS measurement (*gps_lon*,*gps_lat*) taken during surveying and calculated post factum match or not.

	2006	2009	2012	2015	2018
**Match**	100.00	96.32	97.92	99.08	99.77
**No match**	0.00	3.68	2.08	0.92	0.23

In addition to the theoretical grid and survey point location, this data descriptor provides the East-facing transect geo-location data. No additional geo-located spatial information is collected in the transect module and this is probably a shortcoming in the survey design resulting from trade-offs between the cost of the survey and its objectives. The theoretical transect line (with the same geometry as the one provided with this data descriptor) is displayed on the ground document of the surveyor. The surveyor has then to walk on the line and to record the successive land cover and landscape elements as described in the survey methodology. The only geo-location accuracy information relevant for the transect module is thus the same as presented previously, i.e. distance between the theoretical point and the GPS measured surveyed point. Then the successive land covers and landscapes surveyed along the 250-m line are collected as a sequence without distance or geo-located information.

## Usage Notes

To summarize, the work documented in this data descriptor consists of^49^: (1) Multi-year harmonised LUCAS table, (2) Archive with high resolution LUCAS photos, (3) LUCAS survey geometries and point locations, (4) R package^51^, (5) Data descriptor of resulting database and (6) a Documentation table for users to quickly identify the differences of collected data between LUCAS campaigns micro-data and harmonised database.

The harmonised LUCAS product reduces the complexity and layered nature of the original LUCAS datasets. In doing so, it valorizes the effort of many surveyors, data cleaners, statisticians, and database maintainers. The database’s novelty lies in the fact that for the first time, users can query the whole LUCAS archive concurrently, allowing for comparisons and combinations between all variables collected during the relevant reference years. The homogeneity of the product facilitates the unearthing of temporal and spatial relations that were otherwise jeopardized by the physical separation between survey results. Moreover, by avoiding the burden of combing through the cumbersome documentation, the user is now free to concentrate on the research, thereby facilitating scientific discovery and analysis. Naturally, the product suffers from the shortcomings inherent in the source data, such as any inadequate surveying, surveyor or technology-related errors of precision while taking coordinates or measurements, etc. The harmonisation process itself also reveals some inconsistencies in the source data. For instance, certain variables could not be harmonised between survey years. These are mostly related to measurements of percentage or extent of coverage. Where in the early stages of LUCAS surveyors were asked to fill in a multiple choice questionnaire, listing a range of values, in subsequent surveys the surveyor was asked to fill in the actual value in quantified units. This situation applies mostly, though not exclusively, to the 2006 survey, which makes it impossible for these variables to be translated into the user friendly version; therefore in these cases the variables of 2006 must remain in their original coding. Additional information can be found in the comments section of the record descriptor.

Another shortcoming is the change of hierarchy of the LUCAS classification system between the different surveys, mainly concerning LC/LU, as well as LC and LU types. A table is provided to document this shortcoming (see special remarks in the Table (“LC (LU) changes” in the file *LUCAS-Variable_and_Classification* _Changes.xlsx^49^).

## Data Availability

To guarantee transparency and reproducibility, the harmonisation workflow was carried out with open-source tools, namely PostgreSQL (9.5.17)/PostGIS (2.1.8 r13775)) and R (3.4.3)^56^). The code is provided as a R package containing 17 functions along with the documentation on^51^. The LUCAS package includes all the scripts and documentation (also provided in pdf). Additionally, along with the package, a script (*main.R*) builds the harmonised database step by step. The workflow is schematically shown in Fig. 2. All the processing is done with SQL with only column reordering and consistency checks being done in R. The code is freely available under GPL (> = 3) license.

## Online-only Tables

**Online-only Table 1:** Table of added columns.

variable added	to years
gps_altitude	2006
lu1_type	2006, 2009, 2012
lu1_perc	2006, 2009, 2012
lu2_type	2006, 2009, 2012
lu2_perc	2006, 2009, 2012
inspire_plcc1	2006, 2009, 2012
inspire_plcc2	2006, 2009, 2012
inspire_plcc3	2006, 2009, 2012
inspire_plcc4	2006, 2009, 2012
inspire_plcc5	2006, 2009, 2012
inspire_plcc6	2006, 2009, 2012
inspire_plcc7	2006, 2009, 2012
inspire_plcc8	2006, 2009, 2012
nuts3	2006, 2009, 2012, 2015
office_pi	2006, 2009, 2012
ex_ante	2006, 2009, 2012, 2015
car_latitude	2006, 2009, 2012, 2015
car_ew	2006, 2009, 2012, 2015
car_longitude	2006, 2009, 2012, 2015
gps_ew	2009, 2012, 2015
tree_height_maturity	2006, 2009
lm_plough_slope	2006, 2009, 2012, 2015
lm_plough_direct	2006, 2009, 2012, 2015
lm_stone_walls	2006, 2009, 2012, 2015
lm_grass_margins	2006, 2009, 2012, 2015
special_status	2006, 2009
lc_lu_special_remark	2006, 2009
cprn_cando	2006, 2009, 2012, 2015
cprn_lc	2006, 2009, 2012, 2015
cprn_lc1n	2006, 2009, 2012, 2015
cprnc_lc1e	2006, 2009, 2012, 2015
cprnc_lc1s	2006, 2009, 2012, 2015
cprnc_lc1w	2006, 2009, 2012, 2015
cprn_lc1n_brdth	2006, 2009, 2012, 2015
cprn_lc1e_brdth	2006, 2009, 2012, 2015
cprn_lc1s_brdth	2006, 2009, 2012, 2015
cprn_lc1w_brdth	2006, 2009, 2012, 2015
cprn_lc1n_next	2006, 2009, 2012, 2015
cprn_lc1e_next	2006, 2009, 2012, 2015
cprn_lc1s_next	2006, 2009, 2012, 2015
cprn_lc1w_next	2006, 2009, 2012, 2015
cprn_urban	2006, 2009, 2012, 2015
cprn_impervious_perc	2006, 2009, 2012, 2015
eunis_complex	2006, 2009, 2012, 2015
grassland_sample	2006, 2009, 2012, 2015
grass_cando	2006, 2009, 2012, 2015
erosion_cando	2006, 2009, 2012, 2015
bio_sample	2006, 2009, 2012, 2015
soil_bio_taken	2006, 2009, 2012, 2015
bulk0_10_sample	2006, 2009, 2012, 2015
soil_blk_0_10_taken	2006, 2009, 2012, 2015
bulk10_20_sample	2006, 2009, 2012, 2015
soil_blk_10_20_taken	2006, 2009, 2012, 2015
bulk20_30_sample	2006, 2009, 2012, 2015
soil_blk_20_30_taken	2006, 2009, 2012, 2015
standard_sample	2006, 2009, 2012, 2015
soil_std_taken	2006, 2009, 2012, 2015
organic_sample	2006, 2009, 2012, 2015
soil_org_depth_cando	2006, 2009, 2012, 2015
crop_residues	2006, 2009, 2012, 2015
obs_radius	2018
soil_taken	2018
soil_crop	2018
transect	2006, 2018
letter_group	ALL
file_path_thumb_n	ALL
file_path_thumb_s	ALL
file_path_thumb_w	ALL
file_path_thumb_e	ALL
file_path_thumb_p	ALL
year	ALL

**Online-only Table 2:** Table of re-coded variables.

Variable	Year	Old coding	Old label	Interim	Harmonized coding	Harmonized label
lc1/2	2006	C11	Broadleaved forest		C10	Broadleaved woodland
lc1/2	2006	C12	Coniferous forest		C20	Coniferous woodland
lc1/2	2006	C13	Mixed forest		C30	Mixed woodland
lc1/2	2006	D01	Shrubland with sparse tree cover		D10	Shrubland with sparse tree cover
lc1/2	2006	D02	Shrubland without tree cover		D20	Shrubland without tree cover
lc1/2	2006	E01	Grassland with sparse tree/shrub cover		E10	Grassland with sparse tree/shrub cover
lc1/2	2006	E02	Grassland without tree/shrub cover		E20	Grassland without tree/shrub cover
lc1/2	2006	G01	Inland water bodies		G10	Inland water bodies
lc1/2	2006	G02	Inland running water		G20	Inland running water
lc1/2	2006	G03	Coastal water bodies		G30	Transitional water bodies
lc1/2	2006	G05	Glaciers		G50	Glaciers, permanent snow
lu1/2	2006	U400	Abandoned		U410	Abandoned areas
lu1/2	2009	U400	Abandoned		U410	Abandoned areas
soil_taken	2009	3	Point not in soil sample		4	No sample required
lc1/2_perc	2012	2	5 – 10		1	<10%
lc1/2_perc	2012	3	10 – 25		2	10 – 25%
lc1/2_perc	2012	4	25 – 50		3	25 – 50%
lc1/2_perc	2012	5	50 – 75		4	50 – 75%
lc1/2_perc	2012	6	>75		5	>75%
lc_lu_special_remark	2012	1	Tilled/Sowed	100	2	Tilled/sowed
lc_lu_special_remark	2012	2	Harvested field	200	1	Harvested field
lc_lu_special_remark	2012	7	No remark	700	10	No remark
lc_lu_special_remark	2012	8	Not relevant	800	88	Not relevant
obs_type	2015	5	Marine sea		8	Marine sea
obs_type	2015	6	Out of national territory		5	Out of national territory
lc_lu_special_remark	2015	1	Tilled/Sowed	100	2	Tilled/Showed
lc_lu_special_remark	2015	2	Harvested field	200	1	Harvested field
lc_lu_special_remark	2015	7	No remark	700	10	No remark
lc_lu_special_remark	2015	8	Not relevant	800	88	Not relevant
lc_lu_special_remark	2015	9	Temporarily dry	900	8	Temporarily dry
lc_lu_special_remark	2015	10	Temp flooded	1000	9	Temp flooded
lc1/2_perc	2015	2	5 – 10		1	<10%
lc1/2_perc	2015	3	10 – 25		2	10 – 25%
lc1/2_perc	2015	4	25 – 50		3	25 – 50%
lc1/2_perc	2015	5	50 – 75		4	50 – 75%
lc1/2_perc	2015	6	75 – 90		5	>75%
lc1/2_perc	2015	7	>90		5	>75%
osb_type	2018	6	Out of EU28		5	Out of national territory
parcel_area_ha	2018	2	0.1 – 0.5		1	<0.5
parcel_area_ha	2018	3	0.5 – 1		2	0.5 – 1
parcel_area_ha	2018	4	1 – 10		3	1 – 10
parcel_area_ha	2018	5	>10		4	>10
lc1/2_perc	2018	<10			1	<10%
lc1/2_perc	2018	> = 10: <25			2	10–25%
lc1/2_perc	2018	> = 25: <50			3	25–50%
lc1/2_perc	2018	> = 50: <75			4	50–75%
lc1/2_perc	2018	> = 75			5	>75%
lu1/2_perc	2018	<5			1	<5%
lu1/2_perc	2018	> = 5: <10			2	5–10%
lu1/2_perc	2018	> = 10: <25			3	10–25%
lu1/2_perc	2018	> = 25: <50			4	25–50%
lu1/2_perc	2018	> = 50: <75			5	50–75%
lu1/2_perc	2018	> = 75: <90			6	75–90%
lu1/2_perc	2018	> = 90			7	>90%
soil_stones_perc	2018	<10			1	<10%
soil_stones_perc	2018	> = 10: <25			2	10–25%
soil_stones_perc	2018	> = 25: <50			3	25–50%
soil_stones_perc	2018	> = 50			4	>50%

**Online-only Table 3:** Data descriptor of the resulting database.

	variable	type	NR	values	documentation	Description	Comments	collection year
1	point_id	INTEGER	—	—	C1(Instructions), p.141, sec. 9.1.1	Unique point identifier		all
2	nuts0	VARCHAR(2)	—	—	—	NUTS 2016 Level 0		all
3	nuts1	VARCHAR(3)	—	—	—	NUTS 2016 Level 1		all
4	nuts2	VARCHAR(4)	—	—	—	NUTS 2016 Level 2		all
5	nuts3	VARCHAR(5)	—	—	—	NUTS 2016 Level 3		2018
6	th_lat	DECIMAL	88.888888	—	—	Theoretical latitude (WGS84) of the LUCAS point according to the LUCAS grid		all
7	th_long	DECIMAL	88.888888	—	—	Theoretical longitude (WGS84) of the LUCAS point according to the LUCAS grid		all
8	office_pi	INTEGER	—	0 - No; 1 - Yes;	C1(Instructions), p.141, sec. 9.1.1	Indication of whether photo-interpretation has happened in the office for this LUCAS point		2015 2018
9	ex_ante	INTEGER	—	0 - No; 1 - Yes;	C1(Instructions), p.141, sec. 9.1.1			2018
10	survey_date	DATE	—	—		Date on which the survey was carried out		all
11	car_latitude	DECIMAL		—	C1(Instructions), p.141, sec. 9.1.2	Latitude (WGS84) on which the car was parked		2018
12	car_ew	INTEGER		1 - East; 2 - West; 8 - Not relevant;		GPS Car parking East/West		2018
13	car_longitude	DECIMAL		—	C1(Instructions), p.142, sec. 9.1.2	Longitude (WGS84) on which the car was parked		2018
14	gps_proj	INTEGER		1 - WGS84; 2 - No GPS signal; 8 - Not relevant;	C1(Instructions), p.142, sec. 9.1.2	Normal functioning of GPS using “WGS 84” as coordinate system.		all
15	gps_prec	INTEGER		—	C1(Instructions), p.142, sec. 9.1.2	Indication of average location error as given by GPS receiver (in meters)		all
16	gps_altitude	INTEGER		—	C1(Instructions), p.141, sec. 9.1.1	Elevation in m above sea level		2009 2012 2015 2018
17	gps_lat	DECIMAL		—	C1(Instructions), p.142, sec. 9.1.2	GPS latitude of the location from which observation is actually done (WGS84)		all
18	gps_ew	INTEGER		1 - East; 2 – West; 8 – Not relevant;		East-west encoding setting for GPS.		all
19	gps_long	DECIMAL		—	C1(Instructions), p.142, sec. 9.1.2	GPS longitude of the location from which observation is actually done (WGS84)		all
20	obs_dist	INTEGER	−1	—		Indication of the distance between observation location and the LUCAS point as provided by the GPS receiver (in meters).		all
21	obs_direct	INTEGER	8	1 - On the point; 2 - Look to the North; 3 - Look to the East; 8 - Not relevant;	C1(Instructions), p.144, sec. 9.1.4	1 - On the point Point regularly observed. 2 – To North “Look to the North” rule is applied if the point is located on a boundary/ edge or a small linear feature (<3 m wide). 3 – To East “Look to the East” rule is applied if the point is located on a boundary/edge or a small linear feature (<3 m wide) directed. 8 – Not relevant		all
22	obs_type	INTEGER		1 - *In situ* < 100 m; 2 - *In situ* > 100 m; 3 - *In situ* PI; 4 - *In situ* PI not possible; 5 - Out of national territory; 7 - In office PI; 8 – Marine Sea; (only 2015)	C1(Instructions), p.142-144, sec. 9.1.4	The method of observation for the relevant point.	Find more details check the documentation link.	all
23	lc1	VARCHAR (3)		—	C3(Classification), p.11-69, sec. 2	Coding of land cover according to LUCAS 2018 classification.		all
24	lc1_spec	VARCHAR(4)		—	custom doc – LUCAS_lc1_spec_labels.xls	Coding of land cover species according to LUCAS 2018 classification.		2009 2012 2015 2018
25	lc1_perc	VARCHAR		1 - < 10; 2–10–25; 3–25–50; 4–50–75; 5 - > 75; 8 – Not relevant;		The percentage that the land cover (lc1) takes on the ground.	NB! - This coding applies only for the years 2009 – 2015. For 2018 the number represents the percentage of land-cover (0-100). The variable does not exist for 2006	2009 2012 2015 2018
26	lc2	VARCHAR (3)		—	C3(Classification), p.11-69, sec. 2	Coding of land cover according to LUCAS 2018 classification.		all
27	lc2_spec	VARCHAR (4)		—	custom doc – LUCAS_lc1_spec_labels.xls	Coding of land cover species according to LUCAS 2018 classification.		2009 2012 2015 2018
28	lc2_perc	VARCHAR		1 - < 10; 2–10–25; 3–25–50; 4–50–75; 5 - > 75; 8 – Not relevant;		The percentage that the land cover (lc2) takes on the ground.	NB! - This coding applies only for the years 2009 – 2015. For 2018 the number represents the percentage of land-cover (0-100). The variable does not exist for 2006	2009 2012 2015 2018
29	lu1	VARCHAR (4)		—	C3(Classification), p.70-88, sec. 3	Coding of the land use according to LUCAS LU 2018 classification		all
30	lu1_type	VARCHAR (5)		—	C1(Instructions), p.188, sec. 9.2.5	Coding of the land use types according to LUCAS LU 2018 classification		2015 2018
31	lu1_perc	VARCHAR		1 - < 5; 2–5 – 10; 3–10 – 25; 4–25 – 50; 5–50 – 75; 6–75 – 90; 7 - ≥ 90; 8 – Not Relevant;		The percentage that the land use (lu1) takes on the ground.	NB! - This coding applies only for the year 2015. For 2018 the number represents the percentage of land-cover (0-100). The variable does not exist for 2006, 2009 and 2012;	2015 2018
32	lu2	VARCHAR (4)		—	C3(Classification), p.70–88, sec. 3	Coding of the land use according to LUCAS LU 2018 classification		all
33	lu2_type	VARCHAR (5)		—	C1(Instructions), p.188, sec. 9.2.5	Coding of the land use types according to LUCAS LU 2018 classification		2015 2018
34	lu2_perc	VARCHAR		1 - < 5; 2–5 – 10; 3–10 – 25; 4–25 – 50; 5–50 – 75; 6–75 – 90; 7 - ≥ 90; 8 - Not Relevant;		The percentage that the land use (lu2) takes on the ground.	NB! - This coding applies only for the year 2015. For 2018 the number represents the percentage of land-cover (0-100). The variable does not exist for 2006, 2009 and 2012;	2015 2018
35	parcel_area_ha	INTEGER		1 - < 0.5; 2 - 0.5 – 1; 3 - 1 – 10; 4 - > 10; 8 – Not Relevant;		Size of the surveyed parcel in hectares.		2009 2012 2015 2018
36	tree_height_survey	INTEGER		1 - < 5 m; 2 - > 5 m; 8 - Not relevant; 255 – Not identifiable;	C1(Instructions), p.147, sec. 9.1.6	Height of trees at the moment of survey		2009 2012 2015 2018
37	tree_height_maturity	INTEGER		1 - < 5 m; 2 - > 5 m; 8 - Not relevant; 255 – Not identifiable;	C1(Instructions), p.147, sec. 9.1.6	Height of trees at maturity		2012 2015 2018
38	feature_width	INTEGER		1 - < 20 m; 2 - > 20 m; 8 - Not relevant; 255 – Not identifiable;	C1(Instructions), p.147, sec. 9.1.6	Width of the feature		2009 2012 2015 2018
39	lm_stone_walls	INTEGER		1 - No; 2 - Stone wall not maintained; 3 - Stone wall well maintained; 8 - Not relevant;	C1(Instructions), p.148, sec. 9.1.7	Presence of stone walls on the plot.	NB! - only for 2018	2018
40	crop_residues	INTEGER		1 - Yes; 2 - No; 8 - Not relevant;		Presence of crop residues on the plot		2018
41	lm_grass_margins	INTEGER		1 - No; 2 - < 1 m width; 3 - > 1 m width; 8 - Not relevant;	C1(Instructions), p.148, sec. 9.1.7	Presence of grass margins on the plot.	NB! - only for 2018	2018
42	grazing	INTEGER		1 - Signs of grazing; 2 - No signs of grazing; 8 - Not relevant;	C1(Instructions), p.148, sec. 9.1.8	Signs of grazing on the plot.		2009 2012 2015 2018
43	special_status	INTEGER		1 - Protected; 2 - Hunting; 3 - Protected and hunting; 4 - No special status; 8 - Not relevant;	C1(Instructions), p.149, sec. 9.1.8	Whether the plot is part of any specially regulated area.		2012 2015 2018
44	lc_lu_special_remark	INTEGER		1 - Harvested field; 2 - Tilled/sowed; 3 - Clear cut; 4 - Burnt area; 5 - Fire break; 6 - Nursery; 7 - Dump site; 8 - Temporary dry; 9 - Temporary flooded; 10 - No remark; 88 - Not Relevant;	C1(Instructions), p.149-150, sec. 9.1.8	Any special remarks on the land cover/land use.	Find more details check the documentation link.	2012 2015 2018
45	cprn_cando	INTEGER		1 - Yes; 2 - No; 8 - Not relevant;	C1(Instructions), p.150, sec. 9.1.9	Can you do a Copernicus survey on this point?	NB! - only for 2018	2018
46	cprn_lc	TEXT		—	C3(Classification), p.11–69, sec. 2	The land cover on the Copernicus points according to the classification scheme at level2	NB! - only for 2018	2018
47	cprn_lc1n	INTEGER		—	C1(Instructions), p.150, sec. 9.1.9	The extent (in meters) to which the land cover of the Copernicus point stays the same in direction North	NB! - only for 2018	2018
48	cprnc_lc1e	INTEGER		—	C1(Instructions), p.150, sec. 9.1.9	The extent (in meters) to which the land cover of the Copernicus point stays the same in direction East	NB! - only for 2018	2018
49	cprnc_lc1s	INTEGER		—	C1(Instructions), p.150, sec. 9.1.9	The extent (in meters) to which the land cover of the Copernicus point stays the same in direction South	NB! - only for 2018	2018
50	cprnc_lc1w	INTEGER		—	C1(Instructions), p.150, sec. 9.1.9	The extent (in meters) to which the land cover of the Copernicus point stays the same in direction West	NB! - only for 2018	2018
51	cprn_lc1n_brdth	INTEGER		—	C1(Instructions), p.150, sec. 9.1.9	The breath (in %) to the next Copernicus land cover in direction North	NB! - only for 2018	2018
52	cprn_lc1e_brdth	INTEGER		—	C1(Instructions), p.150, sec. 9.1.9	The breath (in %) to the next Copernicus land cover in direction East	NB! - only for 2018	2018
53	cprn_lc1s_brdth	INTEGER		—	C1(Instructions), p.150, sec. 9.1.9	The breath (in %) to the next Copernicus land cover in direction South	NB! - only for 2018	2018
54	cprn_lc1w_brdth	INTEGER		—	C1(Instructions), p.150, sec. 9.1.9	The breath (in %) to the next Copernicus land cover in direction West	NB! - only for 2018	2018
55	cprn_lc1n_next	TEXT		—	C3(Classification), p.11–69, sec. 2	The next Copernicus land cover (level2) in direction North	NB! - only for 2018	2018
56	cprn_lc1e_next	TEXT		—	C3(Classification), p.11–69, sec. 2	The next Copernicus land cover (level2) in direction East	NB! - only for 2018	2018
57	cprn_lc1s_next	TEXT		—	C3(Classification), p.11–69, sec. 2	The next Copernicus land cover (level2) in direction South	NB! - only for 2018	2018
58	cprn_lc1w_next	TEXT		—	C3(Classification), p.11–69, sec. 2	The next Copernicus land cover (level2) in direction West	NB! - only for 2018	2018
59	cprn_urban	INTEGER		1 - Yes; 2 - No; 8 - Not relevant	C1(Instructions), p.151, sec. 9.1.10.1	Is the Copernicus point located in an urban area.	NB! - only for 2018	2018
60	cprn_impervious_perc	INTEGER		—	C1(Instructions), p.151, sec. 9.1.10.1	Assess the percentage of impervious surfaces	NB! - only for 2018	2018
61	inspire_plcc1	INTEGER		—	C1(Instructions), p.151, sec. 9.1.10.2	Assess the percentage of coniferous trees	NB! - only for 2018	2015 2018
62	inspire_plcc2	INTEGER		—	C1(Instructions), p.151, sec. 9.1.10.2	Assess the percentage of broadleaved trees	NB! - only for 2018	2015 2018
63	inspire_plcc3	INTEGER		—	C1(Instructions), p.151, sec. 9.1.10.2	Assess the percentage of shrubs	NB! - only for 2018	2015 2018
64	inspire_plcc4	INTEGER		—	C1(Instructions), p.151, sec. 9.1.10.2	Assess the percentage of herbaceous plants	NB! - only for 2018	2015 2018
65	inspire_plcc5	INTEGER		—	C1(Instructions), p.151, sec. 9.1.10.2	Assess the percentage of lichens and mosses	NB! - only for 2018	2015 2018
66	inspire_plcc6	INTEGER		—	C1(Instructions), p.151, sec. 9.1.10.2	Assess the percentage of consolidated (bare) surface (e.g. rock outcrops)	NB! - only for 2018	2015 2018
67	inspire_plcc7	INTEGER		—	C1(Instructions), p.151, sec. 9.1.10.2	Assess the percentage of unconsolidated (bare) surface (e.g. sand)	NB! - only for 2018	2015 2018
68	inspire_plcc8	INTEGER		—	C1(Instructions), p.151, sec. 9.1.10.2	Sum of all classes must be 100%. This field covers for the difference, if it exists.	NB! - only for 2018	2015 2018
69	eunis_complex	INTEGER		6 - X06; 9 - X09; 10 - Other; 11 - Unknown; 88 - Not relevant	C1(Instructions), p.152, sec. 9.1.11	EUNIS habitat classification	NB! - only for 2018	2018
70	grassland_sample	INTEGER		0 - TRUE; 1 - False;	C1(Instructions), p.152, sec. 9.1.12	Whether or not the point is part of the grassland module	NB! - only for 2018	2018
71	grass_cando	INTEGER		1 - Yes; 2 - No; 8 - Not relevant;	C1(Instructions), p.152, sec. 9.1.12	Is a grassland survey possible?	NB! - only for 2018	2018
72	wm	INTEGER		1 - Irrigation; 2 - Potential irrigation; 3 - Drainage; 4 - Irrigation and drainage; 5 - No visible water management; 8 - Not relevant;	C1(Instructions), p.162, sec. 9.1.13	What type of water management is present at the point		2009 2012 2015 2018
73	wm_source	INTEGER		1 - Well; 2 - Pond/Lake/Reservoir; 3 - Stream/Canal/Ditch; 4 - Lagoon/Wastewater; 5 - Other/Not Identifiable; 6 - Combo - Pond/Lake/Reservoir + Stream/Canal/Ditch; 8 - Not relevant; 16 - Other/Not Identifiable; 17 - Combo - Other/Not Identifiable + Well; 18 - Combo - Other/Not Identifiable + Pond/Lake/Reservoir; 20 - Combo - Other/Not Identifiable + Stream/Canal/Ditch; 24 - Combo - Other/Not Identifiable + Lagoon/Wastewater;	C1(Instructions), p.163, sec. 9.1.13	What is the source of the irrigation at the point	Combo classes exist only for 2009 and were later discontinued. Find more details check the documentation link.	2009 2012 2015 2018
74	wm_type	INTEGER		1 - Gravity; 2 - Pressure: Sprinkle irrigation; 3 - Pressure: Micro-irrigation; 4 - Gravity/Pressure; 5 - Other/Non identifiable; 6 - Combo - Pressure: Sprinkle irrigation + Pressure: Micro-irrigation; 8 - Not relevant; 9 - Combo - Gravity/Pressure + Gravity; 10 - Combo - Pressure: Sprinkle irrigation + Gravity/Pressure; 12 - Combo - Pressure: Micro-irrigation + Gravity/Pressure; 16 - Other/not identifiable; 17 - Combo - Other/Non identifiable + Gravity; 18 - Combo - Other/Non identifiable + Gravity/Pressure; 24 - Combo - Other/Non identifiable + Gravity/Pressure + Pressure: Micro-irrigation;	C1(Instructions), p.162-163, sec. 9.1.13	The type of irrigation present at the point	Combo classes exist only for 2009 and were later discontinued. Find more details check the documentation link.	2009 2012 2015 2018
75	wm_delivery	INTEGER		1 - Canal; 2 - Ditch; 3 - Pipeline; 4 - Other/Non identifiable; 5 - Other/Non identifiable + Canal; 6 - Combo - Pipeline + Ditch; 8 - Not relevant; 10 - Combo - Other/Non identifiable + Ditch; 12 - Combo - Other/Non identifiable + Pipeline;	C1(Instructions), p.163-164, sec. 9.1.13	The irrigation delivery system at the point	Combo classes exist only for 2009 and were later discontinued. Find more details check the documentation link.	2009 2012 2015 2018
76	erosion_cando	INTEGER		1 - Yes; 2 - No; 8 - Not relevant;	C1(Instructions), p.168, sec. 9.1.15	Indicates whether a point is to be considered for assessing erosion (Yes) or not (No)	NB! - only for 2018	2018
77	soil_stones_perc	INTEGER		1 - < 10; 2 - 10 - 25; 3 - 25 - 50; 4 - > 50; 8 – Not relevant;	C1(Instructions), p.164, sec. 9.1.14.2	Indicate the percentage of stones on the surface (does not include stones covered by soil)	NB! - This coding applies only for the years 2009 – 2015. For 2018 the number represents the percentage of stones on the surface (0-100). The variable does not exist for 2006	2018
78	bio_sample	INTEGER		0 - True; 1 - False		Is the point a biodiversity sample point?	NB! - only for 2018	2018
79	soil_bio_taken	INTEGER		0 - True; 1 - False; 8 - Not relevant;	C1(Instructions)	Was a soil-biodiversity sample taken?		2018
80	bulk0_10_sample	INTEGER		0 - True; 1 - False		Indicates whether a point is to be considered for collecting the bulk density between the given range		2018
81	soil_blk_0_10_taken	INTEGER		1 - Yes; 2 – No; 8 - Not relevant;	C1(Instructions)	Has the soil sample between the given range been taken?		2018
82	bulk10_20_sample	INTEGER		0 - True; 1 - False	C1(Instructions)	Indicates whether a point is to be considered for collecting the bulk density between the given range		2018
83	soil_blk_10_20_taken	INTEGER		1 - Yes; 2 – No; 8 - Not relevant;	C1(Instructions)	Has the soil sample between the given range been taken?		2018
84	bulk20_30_sample	INTEGER		0 - True; 1 - False	C1(Instructions)	Indicates whether a point is to be considered for collecting the bulk density between the given range		2018
85	soil_blk_20_30_taken	INTEGER		1 - Yes; 2 – No; 8 - Not relevant;	C1(Instructions)	Has the soil sample between the given range been taken?		2018
86	standard_sample	INTEGER		0 - True; 1 - False	C1(Instructions)	Is the point is a standard soil point?		2018
87	soil_std_taken	INTEGER		1 - Yes; 2 – No; 8 - Not relevant;	C1(Instructions)	Is the standard soil sample was taken?		2018
88	organic_sample	INTEGER		0 - True; 1 - False	C1(Instructions)	Is the point the point an organic sample point?		2018
89	soil_org_depth_cando	INTEGER		1 - Yes; 2 – No; 8 - Not relevant;	C1(Instructions)	Can depth be evaluated?		2018
90	photo_point	INTEGER		1 - Yes; 2 – No; 8 - Not relevant;	C1(Instructions)	Has a photo on the point been taken?		all
91	photo_north	INTEGER		1 - Yes; 2 – No; 8 - Not relevant;	C1(Instructions)	Has a photo looking north been taken?		all
92	photo_east	INTEGER		1 - Yes; 2 – No; 8 - Not relevant;	C1(Instructions)	Has a photo looking east been taken?		all
93	photo_south	INTEGER		1 - Yes; 2 – No; 8 - Not relevant;	C1(Instructions)	Has a photo looking south been taken?		all
94	photo_west	INTEGER		1 - Yes; 2 – No; 8 - Not relevant;	C1(Instructions)	Has a photo looking west been taken?		all
95	obs_radius	INTEGER		1 – 1.5; 2 - 20; 8 - Not relevant;	C1(Instructions)	The radius of observation – whether the immediate or the extended window of observation is taken under consideration.		all
96	soil_taken	INTEGER		1 – Yes 2 – Not possible 3 – No, already taken 4 – No sample required 8 – Not Relevant	C1(Instructions)	Has a soil sample been taken (before 2018)		2009 2012 2015
97	soil_crop	INTEGER		1 - < 10; 2 - 10 - 25; 3 - 25 - 50; 4 - > 50; 8 - Not relevant;	C1(Instructions)	Percentage of residual crop (only 2015)		2009 2012 2015
98	transect	TEXT		—		The changes in landcover as recorded by the east-facing transect line		2009 2012 2015
99	year	INTEGER		—		Which year the point was surveyed		all
100	letter_group	VARCHAR(1)		—		Which letter group (top tier classification) from C3 does the point belong to		all
101	revisit	INTEGER		—		Number of years in which the point has been surveyed.		all
102	file_path_gisco_north	TEXT		—		File path to north-facing image as stored on ESTAT GISCO sever.		all
103	file_path_gisco_south	TEXT		—		File path to south-facing image as stored on ESTAT GISCO sever.		all
104	file_path_gisco_west	TEXT		—		File path to west-facing image as stored on ESTAT GISCO sever.		all
105	file_path_gisco_east	TEXT		—		File path to east-facing image as stored on ESTAT GISCO sever.		all
106	file_path_gisco_point	TEXT		—		File path to point-facing image as stored on ESTAT GISCO sever.		all

## References

[1] Johnson DM (2019). Using the Landsat archive to map crop cover history across the United States. Remote Sensing of Environment.

[2] Gallego, J. & Delincé, J. The European land use and cover area-frame statistical survey. *Agricultural survey methods* 149–168 (2010).

[3] Bettio, M., Delincé, J., Bruyas, P., Croi, W. & Eiden, G. Area frame surveys: aim, principals and operational surveys. *Building Agri-environmental indicators, focussing on the European Area frame Survey LUCAS* 12–27 (2002).

[4] Gallego FJ (2005). Stratified sampling of satellite images with a systematic grid of points. ISPRS Journal of Photogrammetry and Remote Sensing.

[5] Scarnò, M., Ballin, M., Barcaroli, G. & Masselli, M. Redesign sample for Land Use/Cover Area frame Survey (LUCAS) 2018. *Statistical Working Papers* (2018).

[6] Eurostat. Technical reference document c-1: Instructions for surveyors. https://ec.europa.eu/eurostat/documents/205002/8072634/LUCAS2018-C1-Instructions.pdf (2018).

[7] Eurostat. LUCAS Quality Report 2015, https://ec.europa.eu/eurostat/documents/205002/769457/LUCAS+Quality+Report+2015 (2015).

[8] d’Andrimont, R. *et al*. Lucas copernicus 2018: Earth observation relevant *in-situ* data on land cover throughout the european union. Earth System Science Data Discussions 2020, 1–19 (2020). URL, https://essd.copernicus.org/preprints/essd-2020-178/.

[9] Orgiazzi A, Ballabio C, Panagos P, Jones A, Fernández-Ugalde O (2018). LUCAS Soil, the largest expandable soil dataset for Europe: a review. European Journal of Soil Science.

[10] Gallego J, Bamps C (2008). Using CORINE land cover and the point survey LUCAS for area estimation. International Journal of Applied Earth Observation and Geoinformation.

[11] ESTAT. Technical reference document s1: Stratification guidelines, https://ec.europa.eu/eurostat/documents/205002/7329820/LUCAS2018_S1-StratificationGuidelines_20160523.pdf (2018).

[12] Palmieri, A., Martino, L., Dominici, P. & Kasanko, M. Land cover and land use diversity indicators in LUCAS 2009 data. *Land Quality and Land Use Information in the European Union* 59–68 (2011).

[13] d’Andrimont R (2018). Crowdsourced street-level imagery as a potential source of *in-situ* data for crop monitoring. Land.

[14] Karydas C, Gitas I, Kuntz S, Minakou C (2015). Use of LUCAS LC point database for validating country-scale land cover maps. Remote Sensing.

[15] Mack B, Leinenkugel P, Kuenzer C, Dech S (2017). A semi-automated approach for the generation of a new land use and land cover product for germany based on landsat time-series and lucas *in-situ* data. Remote Sensing Letters.

[16] Close O, Benjamin B, Petit S, Fripiat X, Hallot E (2018). Use of Sentinel-2 and LUCAS Database for the Inventory of Land Use, Land Use Change, and Forestry in Wallonia, Belgium. Land.

[17] Pflugmacher D, Rabe A, Peters M, Hostert P (2019). Mapping pan-European land cover using Landsat spectral-temporal metrics and the European LUCAS survey. Remote Sensing of Environment.

[18] Leinenkugel P, Deck R, Huth J, Ottinger M, Mack B (2019). The Potential of Open Geodata for Automated Large-Scale Land Use and Land Cover Classification. Remote Sensing.

[19] d’Andrimont R (2020). Detecting flowering phenology in oil seed rape parcels with sentinel-1 and-2 time series. Remote Sensing of Environment.

[20] Weigand M, Staab J, Wurm M, Taubenböck H (2020). Spatial and semantic effects of lucas samples on fully automated land use/land cover classification in high-resolution sentinel-2 data. International Journal of Applied Earth Observation and Geoinformation.

[21] Eurostat. Lucas 2018 (land use/cover area frame survey). https://ec.europa.eu/eurostat/en/web/lucas/data/primary-data/2006 (2006).

[22] Eurostat. Lucas 2006 (land use/cover area frame survey). https://ec.europa.eu/eurostat/documents/205002/209869/BE_2006_0.xls (2006).

[23] Eurostat. Lucas 2006 (land use/cover area frame survey). https://ec.europa.eu/eurostat/documents/205002/209869/CZ_2006_0.xls (2006).

[24] Eurostat. Lucas 2006 (land use/cover area frame survey). https://ec.europa.eu/eurostat/documents/205002/209869/DE_2006_0.xls (2006).

[25] Eurostat. Lucas 2006 (land use/cover area frame survey). https://ec.europa.eu/eurostat/documents/205002/209869/ES_2006_0.xls (2006).

[26] Eurostat. Lucas 2006 (land use/cover area frame survey). https://ec.europa.eu/eurostat/documents/205002/209869/FR_2006_0.xls (2006).

[27] Eurostat. Lucas 2006 (land use/cover area frame survey. https://ec.europa.eu/eurostat/documents/205002/209869/IT_2006_0.xls (2006).

[28] Eurostat. Lucas 2006 (land use/cover area frame survey). https://ec.europa.eu/eurostat/documents/205002/209869/LU_2006_0.xls (2006).

[29] Eurostat. Lucas 2006 (land use/cover area frame survey). https://ec.europa.eu/eurostat/documents/205002/209869/HU_2006_0.xls (2006).

[30] Eurostat. Lucas 2006 (land use/cover area frame survey). https://ec.europa.eu/eurostat/documents/205002/209869/NL_2006_0.xls (2006).

[31] Eurostat. Lucas 2006 (land use/cover area frame survey). https://ec.europa.eu/eurostat/documents/205002/209869/PL_2006_0.xls (2006).

[32] Eurostat. Lucas 2006 (land use/cover area frame survey). https://ec.europa.eu/eurostat/documents/205002/209869/SK_2006_0.xls (2006).

[33] Eurostat. Lucas 2009 (land use/cover area frame survey). https://ec.europa.eu/eurostat/en/web/lucas/data/primary-data/2009 (2009).

[34] Eurostat. Lucas 2012 (land use/cover area frame survey). https://ec.europa.eu/eurostat/en/web/lucas/data/primary-data/2012 (2012).

[35] Eurostat. Lucas 2015 (land use/cover area frame survey). https://ec.europa.eu/eurostat/en/web/lucas/data/primary-data/2015 (2015).

[36] Eurostat. Lucas 2018 (land use/cover area frame survey). https://ec.europa.eu/eurostat/en/web/lucas/data/primary-data/2018 (2018).

[37] Eurostat. Contents of the 2006 lucas primary data). https://ec.europa.eu/eurostat/documents/205002/209869/Contents_LUCAS_2006_primary_data.xls (2006).

[38] Eurostat. Contents of the 2009 lucas primary data). https://ec.europa.eu/eurostat/documents/205002/208938/Contents-LUCAS-primary-data-2009-20140618-0.xls (2009).

[39] Eurostat. Contents of the 2012 lucas primary data). https://ec.europa.eu/eurostat/documents/205002/208012/Contents-LUCAS-primary-data-12-20140618-.xls (2012).

[40] Eurostat. Technical reference document c-1: Instructions for surveyors. https://ec.europa.eu/eurostat/documents/205002/209869/LUCAS2006_C1-Instructions_20110204.pdf (2006).

[41] Eurostat. Technical reference document c-1: Instructions for surveyors. https://ec.europa.eu/eurostat/documents/205002/208938/LUCAS+2009+Instructions (2009).

[42] Eurostat. Technical reference document c-1: Instructions for surveyors. https://ec.europa.eu/eurostat/documents/205002/208012/LUCAS2012_C1-InstructionsRevised_20130110b.pdf (2012).

[43] Eurostat. Technical reference document c-3: Classification. https://ec.europa.eu/eurostat/documents/205002/209869/LUCAS2006_C3-Classification_20131004.pdf (2006).

[44] Eurostat. Technical reference document c-3: Classification. https://ec.europa.eu/eurostat/documents/205002/208938/LUCAS2009_C3-Classification_20121004.pdf (2009).

[45] Eurostat. Technical reference document c-3: Classification. https://ec.europa.eu/eurostat/documents/205002/8072634/LUCAS2018-C3-Classification.pdf (2018).

[46] Eurostat. Lucas survey 2015 web csv record descriptor. https://ec.europa.eu/eurostat/documents/205002/6786255/WebCsv_RecordDescriptor20161006.pdf (2016).

[47] Eurostat. Lucas survey 2018 web csv record descriptor. https://ec.europa.eu/eurostat/documents/205002/8072634/LUCAS2018-RecordDescriptor-190611.pdf (2019).

[48] Harvey, P. Exiftool (2013).

[49] d’Andrimont R (2020). European Commission, Joint Research Centre (JRC).

[50] d’Andrimont R (2020). figshare.

[51] Yordanov, M., Martinez, L. & d’Andrimont, R. LUCAS R PAckage. *CRAN repository*, https://cran.r-project.org/web/packages/lucas/index.html (2020).

[52] ESTAT. Land cover and land use, landscape (LUCAS) (lan). https://ec.europa.eu/eurostat/cache/metadata/en/lan_esms.htm (2019).

[53] Eurostat. Technical reference document C-4: Quality Control Procedure, https://ec.europa.eu/eurostat/documents/205002/208938/LUCAS2009_C4-QCProcedures_20090303.pdf (2009).

[54] Eurostat. Technical reference document C-4: Quality Control Procedure, https://ec.europa.eu/eurostat/documents/205002/208012/LUCAS2012_C4-QCProcedures_20120113.pdf (2012).

[55] Eurostat. Technical reference document C-4: Quality Control Procedure. https://ec.europa.eu/eurostat/documents/205002/6786255/LUCAS2015-C4-QCProcedures-20150227.pdf (2015).

[56] Team, R Core and others. R: A language and environment for statistical computing (2013).

